# Free‐Standing Bilayer Metasurface With Physically Separated Phase Modulations for Low‐Crosstalk Polarization Multiplexing

**DOI:** 10.1002/nap2.70368

**Published:** 2026-11-11

**Authors:** Bowen Niu, Qun Dai, Yu Ao, Xuanwei Xu, Yu Qiao, Yanjun Hu, Guofang Fan

**Affiliations:** ^1^ Key Laboratory of All Optical Network and Advanced Telecommunication Network Ministry of Education Institute of Lightwave Technology Beijing Jiaotong University Beijing China; ^2^ School of Integrated Circuits Shanghai Polytechnic University Shanghai China

**Keywords:** metasurfaces, polarization multiplexing, spin‐decoupled

## Abstract

Polarization multiplexing metasurfaces are promising for high‐capacity wavefront manipulation but typically suffer from inter‐channel crosstalk that degrades the fidelity of each polarization channel. This paper presents a free‐standing bilayer spin‐decoupled metasurfaces that achieves almost complete decoupling of two circular polarization channels by physically separating the geometric phase and propagation phase modulations into distinct layers. This design overcomes the parametric constraints of single‐layer metasurfaces and maintains high polarization conversion rate. As a demonstration, we implement a dual‐channel beam shaping metasurfaces. Compared with traditional single‐layer design, our device exhibits significant low crosstalk, with the crosstalk suppression ratio (CSR) of the two channels improved by 5.08 and 6.91 dB respectively, and the efficiency and uniformity have also been improved. The device also maintains low crosstalk operation over a wavelength range of ± 30 nm. In addition, dual‐channel hologram shows substantially improved clarity. This approach offers a robust route toward high‐fidelity polarization multiplexed meta‐devices.

## Graphical abstract

This paper presents a free‐standing bilayer spin‐decoupled metasurfaces that achieves almost complete decoupling of two circular polarization channels by physically separating the geometric phase and propagation phase modulations into distinct layers. This design overcomes the parametric constraints of single‐layer metasurfaces, maintains high polarization conversion rate, and achieves low crosstalk polarization multiplexing.

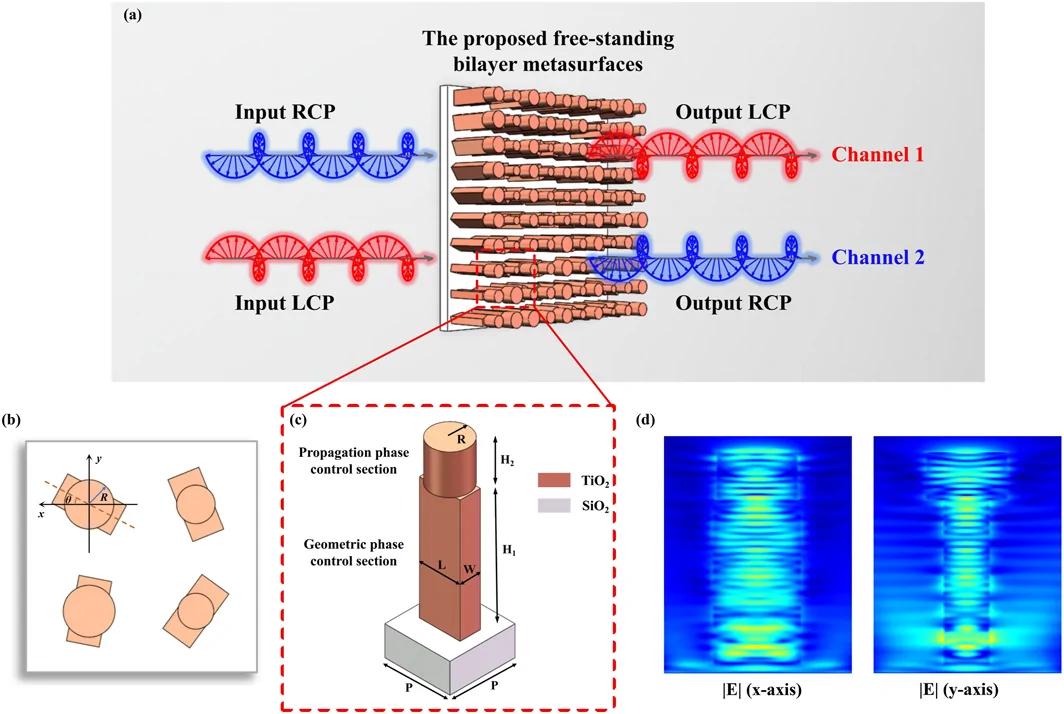

## 1 Introduction

Polarization multiplexing has emerged as a key strategy to enhance the information capacity of optical metasurfaces. Among various approaches, metasurfaces based on the Pancharatnam‐Berry (PB) phase enable efficient wavefront shaping of circularly polarized light simply by rotating anisotropic nanopillars [1, 2]. However, such PB phase metasurfaces suffer from an inherent locked and mirrored constraint, where the wavefront responses for the two circular polarization channels are intrinsically tied as mutual conjugates, preventing independent and arbitrary control over each channel [3, 4]. To achieve complete decoupling of the two orthogonal circular polarization channels, researchers have introduced hybrid strategy that combines geometric phase with propagation phase. By engineering anisotropic nanopillars with tailored dimensions, the propagation phase can be adjusted within the range of 0–2π, while the geometric phase is concurrently imparted through nanopillar rotation. This synergistic approach enables independent wavefront manipulation for left circularly polarized (LCP) and right circularly polarized (RCP) incident light [5, 6]. Following this principle, a variety of spin‐decoupled multifunctional metasurfaces devices have been successfully demonstrated, including vortex beam generation [7], holograms [8], and optical imaging [9].

However, such spin‐decoupled metasurfaces often suffer from inter‐channel crosstalk in practical applications. This crosstalk is mainly manifested as the presence of some intensity components from its orthogonal polarization channel in the optical field of an output channel. As a result, the required intensity distribution is distorted, image resolution is reduced, and the effectiveness of polarization multiplexing is compromised [10, 11]. The main cause of this crosstalk lies in the design of traditional single‐layer spin‐decoupled metasurfaces, where each rectangular nanopillar must handle two independent tasks at the same time: propagation phase control (by dimension) and geometric phase control (by rotation angle). For any given target phase pair for the LCP and RCP channels, the required propagation phase and geometric phase cannot always be perfectly achieved by a physically feasible nanopillar. Therefore, approximations or discrete choices are often made in practice, which prevent the two target phase profiles from being accurately realized. Another important reason for crosstalk is incomplete polarization conversion. In an ideal case, incident circularly polarized light should be fully converted to the orthogonal polarized light after passing through an anisotropic nanopillar. However, in traditional spin‐decoupled designs, the nanopillar dimensions must vary across the metasurfaces to achieve the desired propagation phase modulation. This variation inevitably leads to non‐uniform polarization conversion rate among different meta‐atoms, causing incomplete polarization conversion and thus further contributing to crosstalk.

The crosstalk described above is essentially a consequence of incomplete decoupling. To tackle this issue, various methods have been proposed. One common approach is the interleaved arrangement of meta‐atoms. For example, Liu et al. [12] reported a single‐layer all‐dielectric metasurfaces in which each superpixel consists of two interleaved TiO_2_ nanopillars with independently optimized dimensions. By analytically solving the Jones matrix, the scheme maps the target complex‐amplitude distributions of two orthogonal polarization channels onto the propagation phases and rotation angles of the two nanopillars, achieving independent amplitude and phase control with low crosstalk. However, this superpixel‐based approach reduces spatial resolution. In addition, the interleaved arrangement introduces complex near‐field coupling between adjacent meta‐atoms with different sizes and angles, which may cause unpredictable errors. Another effective method is global optimization without interleaving. Wang et al. [13] reported a non‐interleaved geometric phase metasurfaces in which all meta‐atoms have identical dimensions, and only the rotation angles are globally optimized to achieve low crosstalk circular polarization multiplexing. By using uniform anisotropic meta‐atoms with 99.99% polarization conversion efficiency, they demonstrated low crosstalk holography for both LCP and RCP channels. This design suppresses near‐field coupling by eliminating dimensional variations among meta‐atoms. However, it relies solely on geometric phase control and completely abandons propagation phase modulation, thus losing one degree of freedom. Consequently, the phase responses of the two channels are not truly independent but remain locked to each other.

In recent years, advances in multi‐step nanofabrication techniques have spurred interest in vertically integrated metasurfaces, including multilayer [14], cascaded [15], and double‐sided [16] designs. By distributing complex optical functions across multiple layers, these approaches overcome the limitations of single‐layer designs and simplify the requirements for each individual layer, thereby not only improving optical performance but also expanding functionality [17, 18]. Among these, free‐standing bilayer metasurfaces represent a relatively novel design, consisting of two directly stacked meta‐atoms surrounded by air [19]. This configuration avoids the low refractive‐index contrast often encountered between nanostructures and cladding materials, as well as the optical losses and unwanted reflections caused by gaps in cascaded structures. Although such bilayer designs are generally considered challenging to fabricate, feasible methods have been proven, such as the fabrication process proposed by Dorrah et al. [20] Currently, free‐standing bilayer metasurfaces are mostly used to increase the number of polarization‐multiplexed channels for enhanced functionality [21]. However, simply expanding the number of channels without addressing inter‐channel crosstalk undermines the effectiveness of multifunctionality. In fact, the structural characteristics of free‐standing bilayer metasurfaces also offer a new pathway toward achieving lower crosstalk polarization decoupling.

Here, we propose a free‐standing bilayer metasurfaces design to achieve complete decoupling of two orthogonal circular polarization by physically separating the geometric phase and propagation phase controls into different layers. Specifically, the bottom layer consists of rectangular nanopillars with uniform dimensions, providing geometric phase solely through varying rotation angles. The top layer comprises cylindrical nanopillars with varying radii, utilizing their effective refractive index that varies with radius to induce the accumulation of propagation phase insensitive to circular polarization. As an example, we designed a dual‐channel beam shaping metasurfaces based on this scheme, and determined the structural parameters that can stably achieve high polarization conversion rate and the required propagation phase shifts through parameter scanning. Simulation results show that compared with traditional single‐layer spin‐decoupled metasurfaces, our design achieves low crosstalk and high‐uniformity dual‐channel beam shaping. We analyzed the multi‐wavelength performance of the metasurfaces and examined the impact of layer‐to‐layer misalignment on its working performance. In addition, we also verified the dual‐channel holograms of the metasurfaces.

## 2 Principle

The working principle of the proposed free‐standing bilayer metasurfaces is illustrated in Figure 1a. It independently manipulates the two incident circular polarizations (LCP and RCP), and the output light is converted to the orthogonal polarization, thereby enabling dual‐channel operation. Unlike traditional single‐layer PB phase metasurfaces that require simultaneous variation of both the dimensions and rotation angles of rectangular nanopillars, the meta‐atom of our design is shown in Figure 1c. It consists of a bottom layer for geometric phase control and a top layer for propagation phase control. The bottom layer is composed of rectangular nanopillars with fixed dimensions *W* and *L* across the entire metasurfaces, providing geometric phase solely through rotation angle *θ*. The top layer is composed of cylindrical nanopillars, providing propagation phase modulation through radius *R* variation, as shown in the top view in Figure 1b. The physical mechanism of phase modulation achieved by changing the radius of cylindrical nanopillar is explained in Supporting Information S1: Section S2. The heights *H*_1_ and *H*_2_ of both layers are fixed values, allowing fabrication by two‐layer processing. The substrate material is SiO_2_, and the nanopillars material is TiO_2_.

**FIGURE 1:**
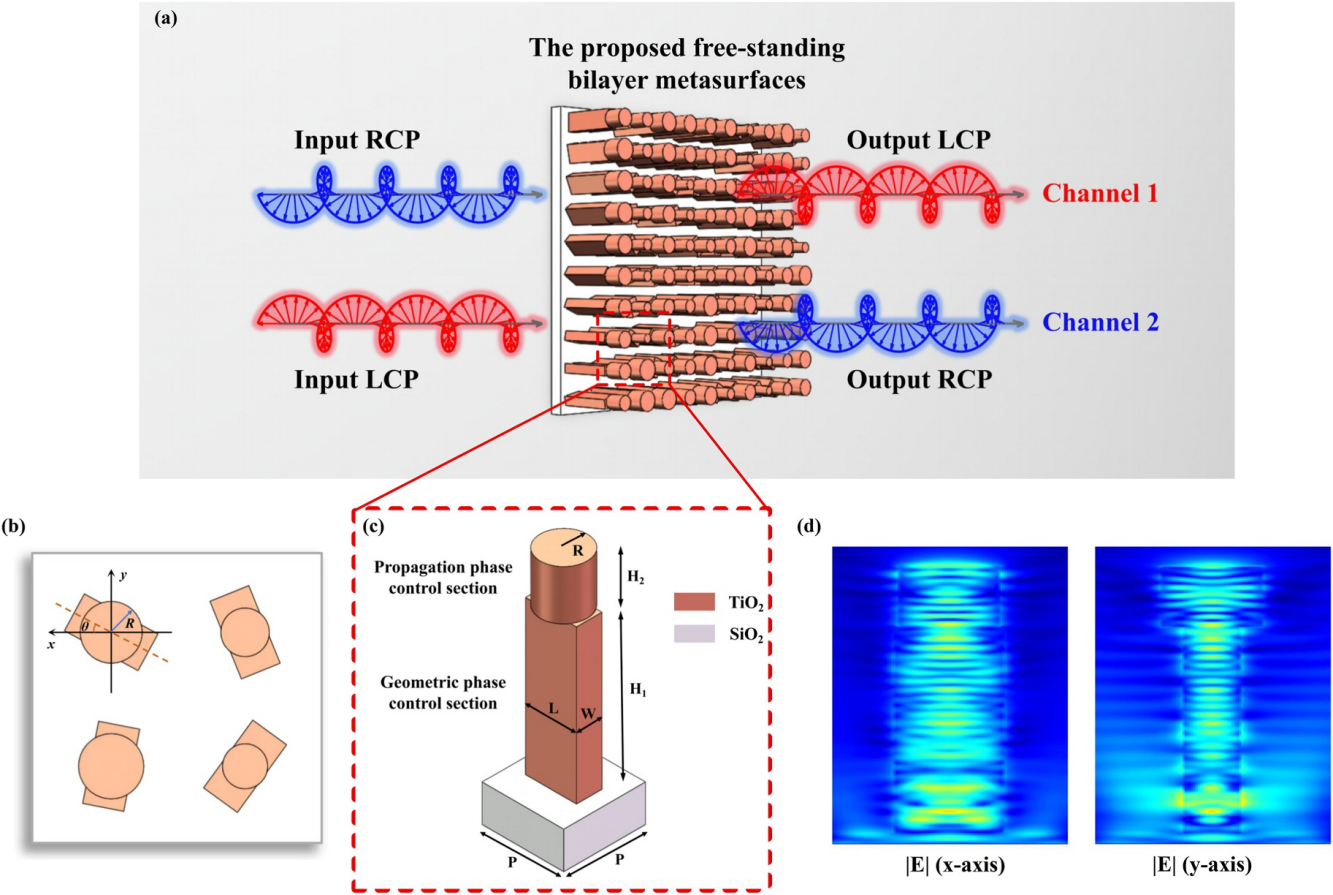
(a) Schematic diagram of the proposed free‐standing bilayer metasurfaces, which enables dual‐channel low crosstalk polarization multiplexing controlled by the incident circular polarization. (b) Top view of the metasurfaces. (c) Meta‐atom structure, consisting of a bottom layer rectangular nanopillars serving as the geometric phase control section (height = *H*_1_) and a top layer cylindrical nanopillars serving as the propagation phase control section (height = *H*_2_). (d) Cross‐sectional total electric field magnitude of the meta‐atom.

This free‐standing bilayer design physically separates the two phase modulation mechanisms, eliminating the parametric degeneracy inherent in single‐layer design. For the bottom layer, since all rectangular nanopillars share identical dimensions, the polarization conversion rate can be optimized to be both high and uniform across the entire metasurfaces, introducing almost no unintended amplitude or phase modulation. For the top layer, the isotropic cylindrical nanopillars introduce no polarization‐dependent response, providing identical phase delays for both LCP and RCP light, which reduces cross‐coupling. Moreover, this combination of a uniform‐dimension layer and an isotropic layer also simplifies near‐field coupling effects. With this design, we aim to achieve low crosstalk dual‐channel independent beam manipulation, offering a new solution for improving the performance of polarization‐multiplexed metasurfaces devices. Figure 1d shows the cross‐sectional total electric field magnitude of the proposed free‐standing bilayer metasurfaces along the *x* and *y*‐directions.

To determine the appropriate parameters for the proposed free‐standing bilayer metasurfaces, we analyzed the parameters of the meta‐atom, with the results shown in Figure 2. Here we fix the period of the meta‐atom as *P* = 600 nm, the total height *H*_1_ + *H*_2_ = 4.5 μm, and the rectangular nanopillars dimensions *W* = 250 nm, *L* = 500 nm. Using 532 nm wavelength LCP beam as the incident. Figure 2a,b respectively show the polarization conversion rate for the output RCP and the overall propagation phase under different height ratios *H*_*1*_/*H*_2_ and cylinder radii *R*. It should be noted that the simulation is based on the finite difference time domain (FDTD) method, using periodic boundary conditions in the *x* and *y* directions and PML boundary conditions in the *z* direction. The mesh size is 10 nm × 10 nm × 10 nm to ensure sufficient spatial resolution within the meta‐atom structure. The complex refractive indices of TiO_2_ and SiO_2_ are taken from material 3D sample data. Combine two plane wave sources with polarization angles and phases that are 90° apart, and incident them along the *z* direction to simulate circularly polarized incident beam, with amplitude normalized to 1. Simulate diffraction propagation results by projecting near‐field transmission data onto far‐field.

**FIGURE 2:**
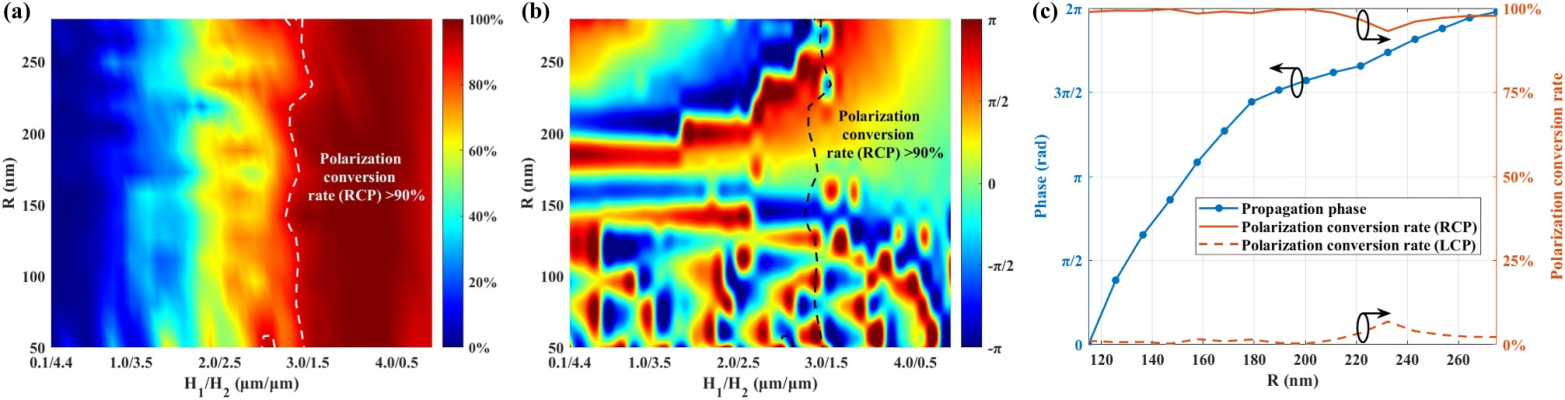
The parameter scanning results of the height ratios *H*_1_/*H*_2_ and radii *R* of the free‐standing bilayer meta‐atom. (a) The polarization conversion rate of RCP. (b) Propagation phase. (c) The polarization conversion rate and the propagation phase versus *R* when *H*_1_ = 3.4 μm and *H*_2_ = 1.1 μm.

First, from Figure 2a, it can be seen that when the height *H*_1_ is relatively low, the bottom layer is too thin to achieve ideal polarization conversion. Therefore, the bottom layer must have sufficient height. The region where the polarization conversion rate exceeds 90% is marked in Figure 2a. This region is then mapped onto Figure 2b, from which we select the structural parameters that can provide the desired propagation phase variation. Ultimately, we chose meta‐atoms with *H*_1_ = 3.4 μm, *H*_2_ = 1.1 μm, and *R* ranging from 115 to 275 nm. Under these parameters, the polarization conversion rate and the propagation phase versus cylindrical nanopillar radius *R* are shown in Figure 2c. It can be seen that over the entire range of *R*, the meta‐atoms provide a full 2π propagation phase shift, while the polarization conversion rate for the RCP output remains stably above 90%. This stable performance benefits from the structural advantages of the free‐standing bilayer design. The determined structure parameters are shown in Table 1.

**TABLE 1:** The structure parameters of the meta‐atoms.

Parameter	Values
*P*	600 nm
*W*	250 nm
*L*	500 nm
*H*_1_	3.4 μm
*H*_2_	1.1 μm
*R*	115–275 nm

To verify the decoupling between the two layers of the proposed bilayer metasurfaces, we took the incident LCP beam as an example and simulated the transmission phase variation and polarization conversion rate of the output orthogonal polarization beam under all selected bilayer structure parameter combinations, as shown in the Figure 3.

**FIGURE 3:**
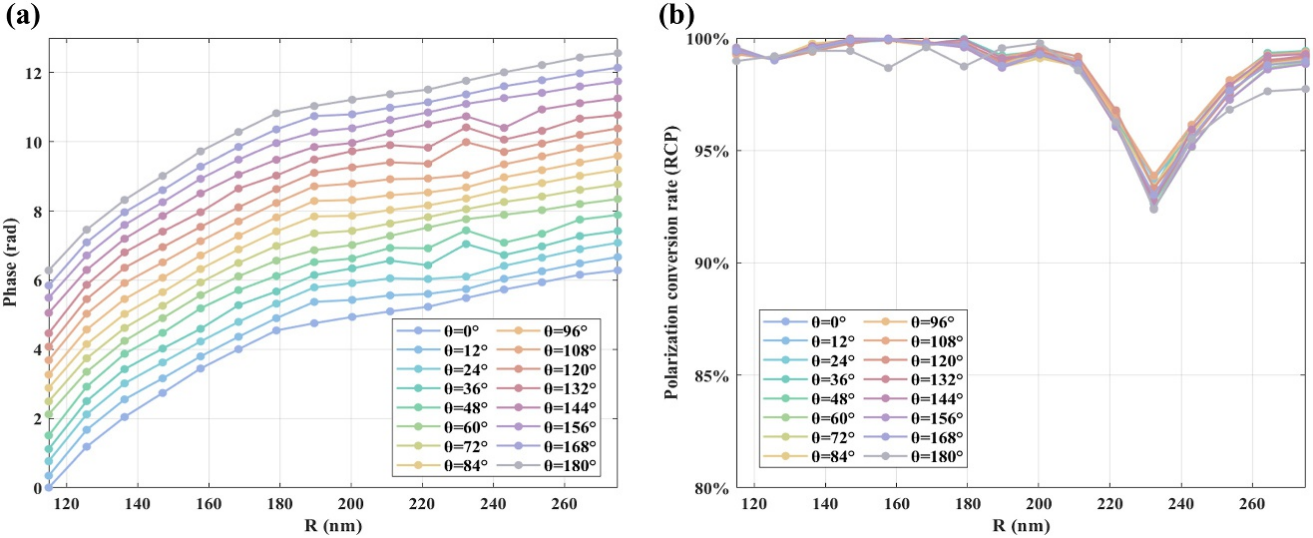
(a) The phase variation of the output orthogonal polarization beam under all bilayer structure parameter combinations. (b) The polarization conversion rate of the output orthogonal polarization beam under all bilayer structure parameter combinations.

From Figure 3a, it can be seen that for the purity of propagation phase control (i.e. the independence of *R*), at any fixed rectangular nanopillar rotation angle *θ*, changing the cylindrical nanopillar radius *R* can cover a range of 2π phase shift in the orthogonal polarization, and the shape of these curves hardly changes with *θ*. This proves that the modulation of propagation phase is almost entirely dominated by *R* and is not affected by the rotation state of the bottom layer rectangular nanopillars. For the purity of geometric phase control (i.e., the independence of *θ*), at any fixed *R*, the variation of phase with *θ* almost follows a linear relationship of 2*θ*. This proves that the introduction of geometric phase can also be regarded as independent of the top layer cylindrical nanopillars. Furthermore, as shown in Figure 3b, the polarization conversion rate remains above 90% for all combinations of *R* and *θ*. This proves the stability of the polarization conversion of the proposed structure. In summary, the two layers of the proposed metasurfaces can be regarded as having approximately independent phase control, and therefore can be reasonably considered as approximately decoupled, with its complex Jones response approximately satisfying

(1) $$J=\left[\begin{array}{cc} t_{\text{LL}} & t_{\text{LR}}\\ t_{\text{RL}} & t_{\text{RR}} \end{array}\right]\approx \left[\begin{array}{cc} 0 & A_{\text{LR}}\left(R,\theta \right)\cdot e^{i\left[\varphi \left(R\right)+2\theta \right]}\\ {A_{\text{RL}}\left(R,\theta \right)\cdot e}^{i\left[\varphi \left(R\right)‒2\theta \right]} & 0 \end{array}\right],$$

where *φ*(*R*) is the propagation phase controlled by the cylindrical nanopillar radius *R*. $A_{\text{LR}}\left(R,\theta \right)$ and $A_{\text{RL}}\left(R,\theta \right)$ represent the transmission amplitudes, and their corresponding polarization conversion rates remain above 90%.

We also compared the phase response *φ*_measured_ of the measured complex transmission coefficients of LCP → RCP and RCP → LCP channels under all bilayer structure parameter combinations with the ideal decoupling model *φ*_ideal_ *= φ*(*R*) *±* 2*θ*. Among them, the phase shift curve at *θ* = 0° is taken as the reference for *φ*(*R*). We calculated the phase deviation based on Δ*φ* = |*φ*_measured_ − *φ*_ideal_|. Table 2 shows the analysis values of phase deviation and polarization conversion rate of all bilayer meta‐atoms in two channels. Including average phase deviation, maximum phase deviation, RMS phase deviation, as well as the average, maximum, and minimum values of polarization conversion rate.

**TABLE 2:** The analysis values of phase deviation and polarization conversion rate of all meta‐atoms.

Analysis values	LCP→RCP	RCP→LCP
Average phase deviation (rad)	0.0781	0.0944
Maximum phase deviation (rad)	0.3521	0.3823
RMS phase deviation (rad)	0.1064	0.1388
Average polarization conversion rate (%)	98.42	98.70
Maximum polarization conversion rate (%)	99.99	99.99
Minimum polarization conversion rate (%)	92.38	92.44

The results indicate that the average phase deviation of the LCP → RCP channel is only 0.0781 rad, and the average phase deviation of the RCP → LCP channel is only 0.0944 rad. Although the maximum phase deviations of the two channels are relatively high, the RMS phase deviations are very close to the average phase deviations. The RMS phase deviation of LCP → RCP channel is 0.1064 rad, and the RCP → LCP channel is 0.1388 rad. This indicates that in the majority of structural parameter combinations, phase deviations are concentrated at lower values, with only a very small number of combinations exhibiting significant deviations. In addition, the average polarization conversion rates of both channels are greater than 98%, and the minimum polarization conversion rates are also greater than 92%. This analysis proves that the linear superposition relationship of the ideal decoupling model is applicable in the entire structure parameter space with minimal deviation. There will be no significant performance degradation due to phase decomposition errors.

In addition, considering the special structure of the proposed free‐standing bilayer metasurfaces, in the case where the radius of the cylindrical nanopillar is large, the cylindrical nanopillars will overhang the supporting rectangular nanopillars. Therefore, it is necessary to discuss the mechanical stability of the free‐standing bilayer structure in this situation. We set the radius of the cylindrical nanopillar to a maximum value of 275 nm, at which point the overhanging part reaches its maximum. We conducted finite element analysis on this structure to evaluate its stress and deformation under gravity. The result is shown in Figure 4.

**FIGURE 4:**
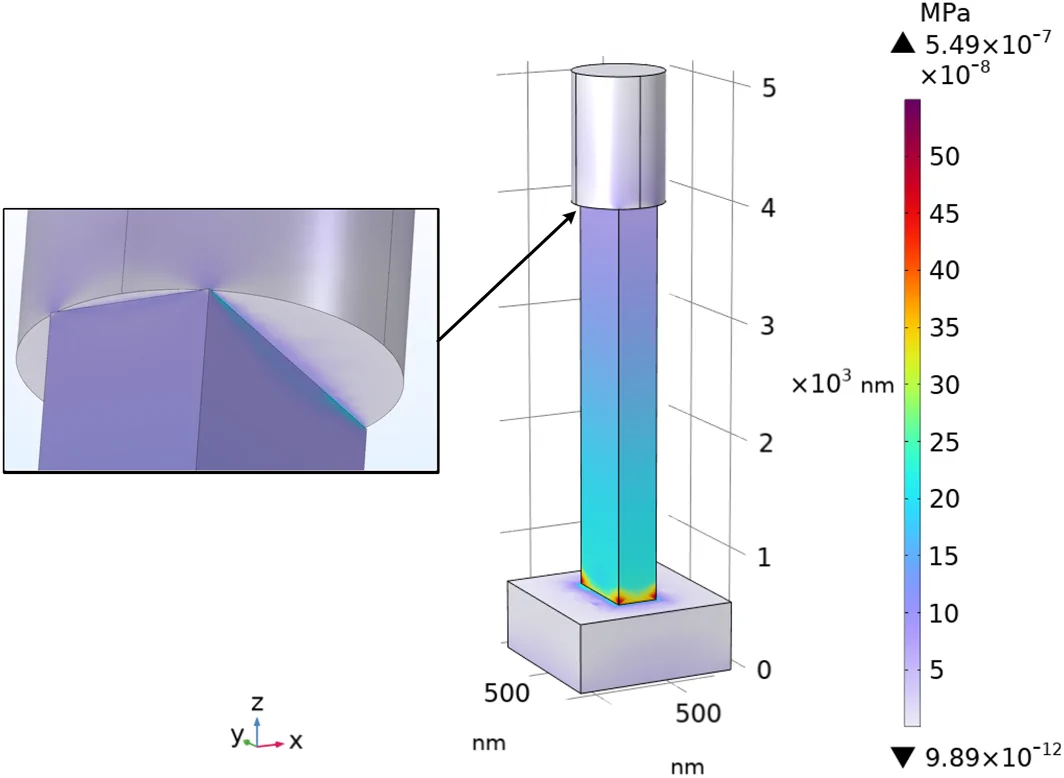
Mechanical stability analysis of free‐standing bilayer metasurfaces structure with top layer cylindrical nanopillar radius of 275 nm.

From the simulation results, it can be seen that in the proposed bilayer structure, the bottom of the rectangular nanopillar will bear the maximum stress, which is only 5.49 × 10^−7^ MPa. This is not enough to cause the TiO_2_ nanostructure to fracture. The overhanging part bears less stress and the deformation can be almost ignored. Therefore, the proposed free‐standing bilayer structure has sufficient mechanical stability.

## 3 Result and Discussion

### 3.1 Free‐Standing Bilayer Metasurfaces for Low Crosstalk Dual‐Channel Beam Shaping

To verify the performance of the proposed free‐standing bilayer metasurfaces, we applied it to dual‐channel beam shaping. As illustrated in Figure 5a, when the incident beam is LCP, the output RCP produces a rectangular beam of 1 × 15 mm at the target plane, with an off‐axis distance of 3 mm along the horizontal direction. When the incident beam is RCP, the output LCP produces a rectangular beam of 15 × 1 mm at the target plane, with an off‐axis distance of 3 mm along the longitudinal direction. Designing the target beams in this manner for the two channels facilitates better analysis of crosstalk effects. The incident wavelength is 532 nm. The required 16‐level phase distributions Phase_1_ and Phase_2_ for the two channels are calculated using IFTA, with a total pixel number of 1500 × 1500, and a working distance of 100 mm. The required geometric phase distribution Phase_*θ*_ and propagation phase distribution Phase_*φ*_ are then calculated using Equations (2) and (3).

(2) $${\text{Phase}}_{\theta }=\frac{{\text{Phase}}_{1}-{\text{Phase}}_{2}}{4},$$

(3) $${\text{Phase}}_{\varphi }=\frac{{\text{Phase}}_{1}+{\text{Phase}}_{2}}{2}.$$

**FIGURE 5:**
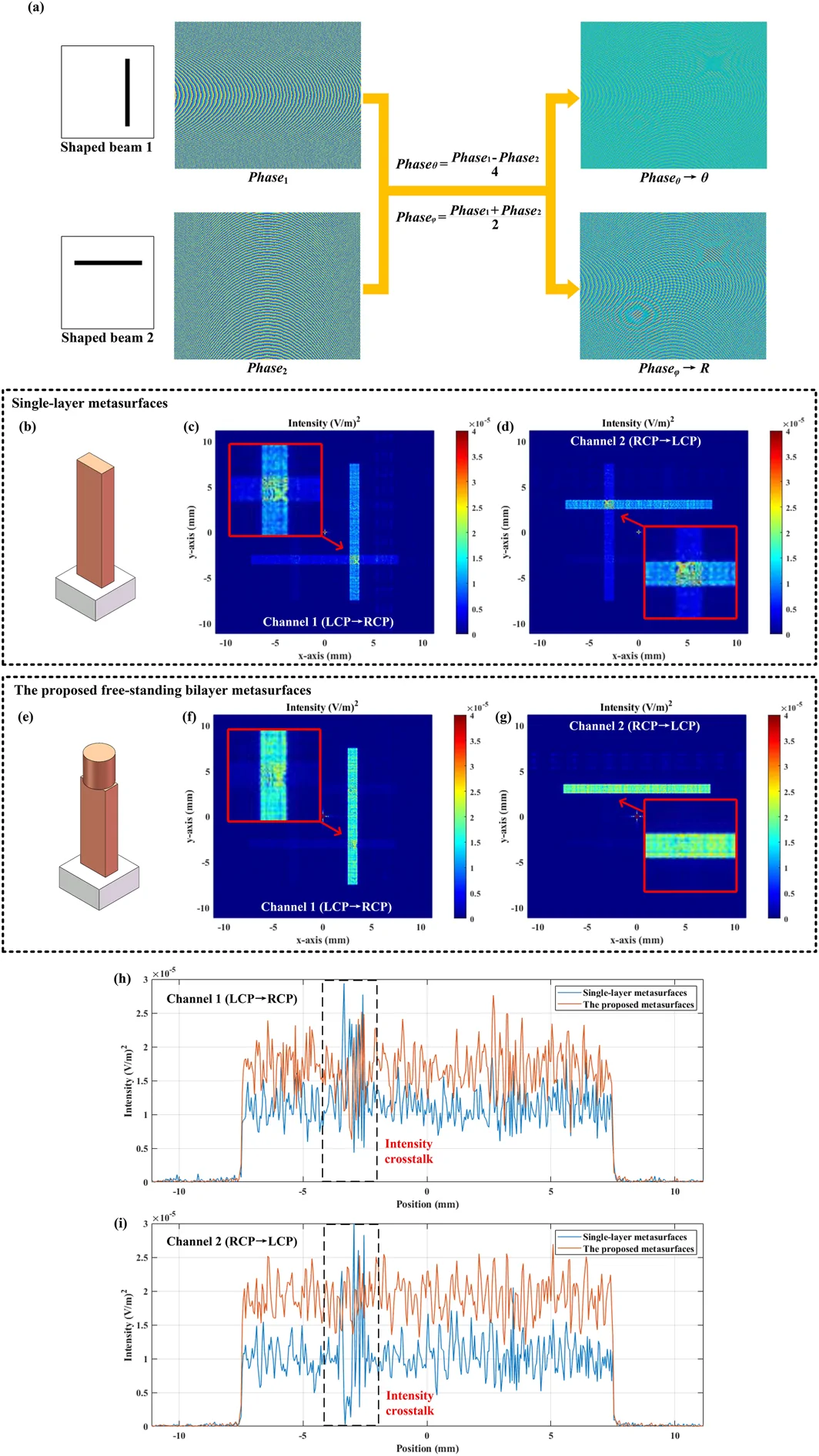
(a) Calculation process of the phases for dual‐channel beam shaping. (b)–(d) The dual‐channel beam shaping achieved by traditional single‐layer spin‐decoupled metasurfaces exhibits significant crosstalk phenomenon. In a certain channel, there is an unexpected conjugate intensity from another channel. (e)–(g) The proposed free‐standing bilayer metasurfaces significantly reduces the crosstalk of dual‐channel beam shaping. Uniform and improved efficiency target beam can be achieved in both channels. (h)–(i) Comparison of cross‐sectional intensity profiles of the shaped beams achieved by two structures in two channels.

The geometric phase distribution corresponds to the rotation angle *θ* of the bottom layer rectangular nanopillars, while the propagation phase distribution is mapped to the radius *R* of the top layer cylindrical nanopillars. We conducted representative heterogeneous‐supercell simulations, as detailed in Supporting Information S1: Section S3, which demonstrated that the simulated periodic independent unit‐cell response is still effective in the assembled metasurfaces. For comparison, a traditional single‐layer spin‐decoupled metasurfaces with the same height of 4.5 μm and period of 600 nm is designed to achieve the same functionality. The meta‐atoms selected by parameter scanning are shown in Supporting Information S1: Section S1. It can be seen that, in order to achieve the desired propagation phase variation, the rectangular nanopillars have non‐uniform dimensions, resulting in inconsistent polarization conversion rates. When these meta‐atoms are assembled into metasurfaces, the overall performance will decrease. The shaped beam quality is evaluated using crosstalk suppression ratio (CSR), coefficient of variation (CV), diffractive efficiency (DE) and absolute efficiency (AE), as follows:

(4) $$\text{CSR}=10\;\log_{10}\frac{P_{\text{signal}}}{P_{\text{crosstalk}}},$$

(5) $$\text{CV}=\frac{\sigma }{\mu },$$

(6) $$\text{DE}=\frac{P_{\text{signal}}}{P_{\text{output}}},$$

(7) $$\text{AE}=\frac{P_{\text{signal}}}{P_{\text{incident}}},$$

where *P*_signal_ is the total power within the target shaped beam signal area, and *P*_crosstalk_ is the power within the conjugate (undesired) area. *P*_output_ represents the total power of the target plane, and *P*_incident_ represents the incident power. *μ* and *σ* represent the average and standard deviation of the intensity within the signal area, respectively. CSR is used to directly measure leakage between channels. CV is used to characterize the uniformity of the shaped beam. Diffractive efficiency is used to measure the energy entering the target shaped beam signal area within this channel. Absolute efficiency is used to evaluate energy loss caused by incomplete polarization conversion, reflection, or other factors.

Figure 5b–d show the dual‐channel performance of the traditional single‐layer spin‐decoupled metasurfaces. In Channel 1 (LCP → RCP), although the rectangular shaped beam is successfully generated at the desired position on the target plane, some unwanted intensity distribution can still be observed. This is the conjugate intensity from the other channel, which causes crosstalk with the target beam in the current channel and leads to local intensity distortion. A magnified view of the crosstalk region is shown in Figure 5c. The CSR of this channel is 3.81 dB. The uniformity of the shaped beam is poor, with a CV of 35.33%. In addition, the presence of conjugated intensity results in lower efficiency, with a diffractive efficiency of 49.05% and an absolute efficiency of 6.90%. The same phenomenon occurs in Channel 2 (RCP → LCP), with the magnified crosstalk region shown in Figure 5d. The CSR of this channel is 3.81 dB. The CV of the shaped beam is 36.55%, the diffractive efficiency is 50.52%, and the absolute efficiency is 7.34% (Table 3).

**TABLE 3:** Comparison of shaped beam quality in dual‐channel between two types of metasurfaces.

Method	Channel 1 (LCP → RCP)				Channel 2 (RCP → LCP)			
	CSR (dB)	CV (%)	DE (%)	AE (%)	CSR (dB)	CV (%)	DE (%)	AE (%)
The proposed metasurfaces	8.89	26.87	60.23	10.76	10.72	23.00	67.24	12.90
Single‐layer metasurfaces	3.81	35.33	49.05	6.90	3.81	36.55	50.52	7.34

Figure 5e–g present the dual‐channel performance of the proposed free‐standing bilayer metasurfaces. As shown in Figure 5f,g, the unwanted conjugate intensity is almost invisible in both Channel 1 and Channel 2, and the target shaped beams exhibit almost no intensity distortion caused by crosstalk. For Channel 1, the CSR is 8.89 dB, which is representing an improvement of 5.08 dB compared with the single‐layer design. The CV is 26.87%, exhibits an improvement of 8.46%. The diffractive efficiency reached 60.23%, exhibits an increase of 11.18%. The absolute efficiency is 10.76%, exhibits an increase of 3.86%. For Channel 2, the CSR is 10.72 dB, exhibits an improvement of 6.91 dB. The CV is 23.00%, exhibits an improvement of 13.55%. The diffractive efficiency is 67.24%, exhibits an improvement of 16.72%. The absolute efficiency is 12.90%, exhibits an improvement of 5.56%. It can be noted that the absolute efficiency is relatively modest, mainly due to power loss caused by reflection and scattering. According to simulation statistics, the average transmittance of the proposed meta‐atom structure is about 25%, which means that about 75% of the incident power will be lost due to reflection and scattering. In addition, secondary reasons include a small amount of power loss caused by incomplete polarization conversion in the transmitted light (Polarization conversion rate > 90%), as well as power loss caused by 16‐level phase quantization. All these power losses together result in a relatively moderate absolute efficiency value. The low transmittance can mainly be attributed to the additional interface reflection introduced by the complex bilayer stacking structure, and the high scattering loss of high‐aspect‐ratio nanopillars. It is expected to further improve the transmittance by introducing the anti‐reflection layer.

Figure 5h,i show the cross‐sectional intensity profiles of the shaped beams achieved by the single‐layer metasurfaces and the free‐standing bilayer metasurfaces in Channel 1 and Channel 2, respectively. It can be seen that the shaped beam of the single‐layer metasurfaces exhibits obvious intensity surges in the crosstalk region, while the intensity in other regions remains relatively weak. In contrast, the shaped beam of the free‐standing bilayer metasurfaces shows no intensity distortion and maintains a higher overall intensity. Therefore, the proposed free‐standing bilayer metasurfaces achieve low crosstalk dual‐channel beam shaping. We also compared the crosstalk and uniformity of the shaped beam under continuous phase and 16‐level discretized phase, as detailed in Supporting Information S1: Section S4. The results indicate that phase quantization has a relatively small impact.

### 3.2 Multi‐Wavelength Performance of the Free‐Standing Bilayer Metasurfaces for Dual‐Channel Beam Shaping

To evaluate the multi‐wavelength performance of the proposed method, we adopted the dual‐channel beam shaping metasurfaces described in Section 3.1. The beam shaping performance was simulated at incident wavelengths of 532 ± 30 nm. Figure 6a shows the intensity distributions of the shaped beams for both Channel 1 and Channel 2 at each incident wavelength. According to diffraction theory, the pixel size at the target plane changes with the incident wavelength. As can be seen from the Figure 6a, when the incident wavelength decreases, the size of the rectangular shaped beam becomes smaller; when the wavelength increases, the beam size becomes larger.

**FIGURE 6:**
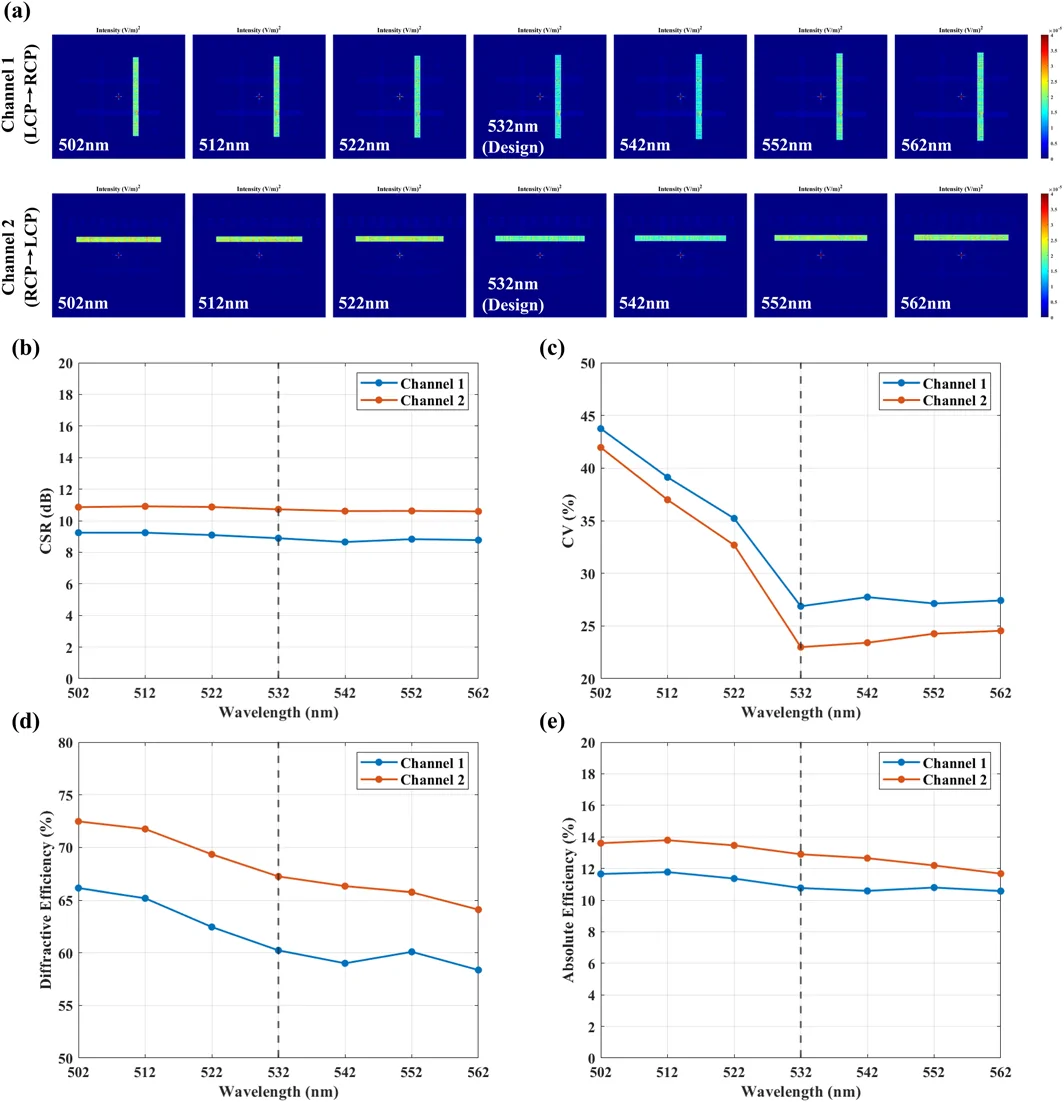
(a) The intensity distributions of the shaped beams in Channel 1 and Channel 2 at 532 ± 30 nm incident wavelength. (b) CSR, (c) CV, (d) diffractive efficiency and (e) absolute efficiency of the shaped beams in Channel 1 and Channel 2 at 532 ± 30 nm incident wavelengths.

Figure 6b shows the calculated CSR at different incident wavelengths for Channel 1 and Channel 2. It can be seen that the crosstalk intensity remains at a low level throughout the entire wavelength range, and the energy stays well confined within the rectangular shaped beam. The value of CSR remains almost unchanged. This indicates that the proposed free‐standing bilayer metasurfaces maintains low crosstalk operation even when the incident wavelength is changed. Figure 6c shows the calculated CV at different incident wavelengths for Channel 1 and Channel 2. Figure 6d,e show the calculated diffractive efficiency and absolute efficiency of two channels at different incident wavelengths, respectively. It should be noted that the size of the signal area remains fixed, while the size of the rectangular shaped beam varies with wavelength. When the wavelength decreases, the shaped beam becomes smaller than the signal area, causing the CV to increase sharply. At the same time, additional energy is included within the signal area, leading to an increase in the calculated efficiency. When the wavelength increases, the shaped beam becomes larger than the signal area, and the signal area essentially covers only a portion of the beam interior. As a result, the efficiency decreases, while the CV increases only slightly, indicating that the uniformity inside the shaped beam remains well preserved.

To further evaluate the multi‐wavelength performance of the proposed free‐standing bilayer metasurfaces, we also simulated the intrinsic performance of the meta‐atoms library under the incident wavelength of 532 ± 30 nm. We calculated the average phase deviation and average polarization conversion rate in two channels. The results are shown in Table 4.

**TABLE 4:** The average phase deviation and polarization conversion rate of all meta‐atoms under the incident wavelength of 532 ± 30 nm.

	Channel	502 nm	512 nm	522 nm	532 nm	542 nm	552 nm	562 nm
Average phase deviation	LCP → RCP	0.2352 rad	0.2191 rad	0.1937 rad	0.0781 rad	0.2329 rad	0.2555 rad	0.2880 rad
	RCP → LCP	0.2434 rad	0.2265 rad	0.2044 rad	0.0944 rad	0.2567 rad	0.2712 rad	0.2878 rad
Average polarization conversion rate	LCP → RCP	95.43%	97.15%	98.03%	98.42%	98.34%	98.40%	97.39%
	RCP → LCP	95.59%	97.42%	98.27%	98.70%	98.48%	98.50%	97.41%

The results indicate that the intrinsic performance of the meta‐atoms library will degrade when deviating from the design wavelength. At the incident wavelength range of 532 ± 30 nm, the average phase deviation of the LCP → RCP channel increase to 0.2880 rad, while the average phase deviation of the RCP → LCP channel increase to 0.2878 rad; The average polarization conversion rate of the two channels does not change much and remains above 95%. Phase deviation is mainly caused by the difference in dispersion response of the cylindrical nanopillars radii when the wavelength deviates from the design value. For a 16‐level discrete phase, a phase deviation of 0.2880 rad is equivalent to approximately 0.7 quantization steps. In far‐field diffraction, this phase deviation usually does not cause catastrophic performance degradation. Overall, the proposed free‐standing bilayer metasurfaces can maintain relatively stable low crosstalk performance within the range of ± 30 nm around the design wavelength.

### 3.3 Analysis of Layer‐to‐Layer Misalignment Errors of the Free‐Standing Bilayer Metasurfaces

Considering that the proposed free‐standing bilayer metasurfaces is prone to layer‐to‐layer misalignment during actual fabricating. We also used the dual‐channel beam shaping metasurfaces described in Section 3.1, taking the incident LCP beam as an example to evaluate its performance under the influence of layer‐to‐layer misalignment. We simulated three types of layer‐to‐layer misalignment: lateral *δx*, longitudinal *δy*, and oblique *δc*, as shown in Figure 7. We calculated the evaluation values for the RCP output shaped beam as the misalignment errors ranged from 0 to 100 nm, and the results are shown in Tables 5, 6, 7.

**FIGURE 7:**
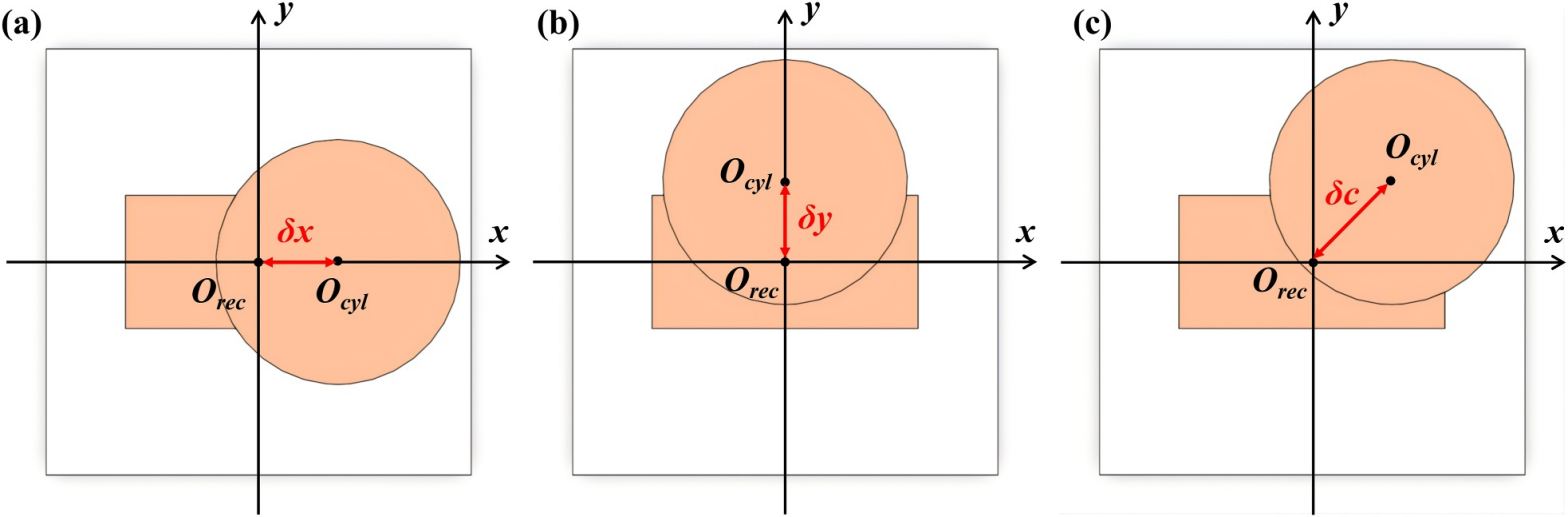
Schematic diagram of three types of layer‐to‐layer misalignment: Lateral, longitudinal, and oblique.

**TABLE 5:** The influence of 0–100 nm lateral layer‐to‐layer misalignment on beam shaping Performance.

Parameter	*δx =* 0 nm	*δx =* 25 nm	*δx =* 50 nm	*δx =* 75 nm	*δx =* 100 nm
CSR (dB)	8.89	8.90	9.01	9.17	9.72
CV (%)	26.87	27.08	27.20	27.74	28.16
DE (%)	60.23	60.18	61.99	63.79	64.54
AE (%)	10.76	10.55	10.36	10.12	8.13

**TABLE 6:** The influence of 0–100 nm longitudinal layer‐to‐layer misalignment on beam shaping performance.

Parameter	*δy =* 0 nm	*δy =* 25 nm	*δy =* 50 nm	*δy =* 75 nm	*δy =* 100 nm
CSR (dB)	8.89	8.91	8.99	9.15	9.70
CV (%)	26.87	27.11	27.31	27.62	28.07
DE (%)	60.23	59.78	60.78	61.25	61.15
AE (%)	10.76	10.47	10.06	9.25	7.42

**TABLE 7:** The influence of 0–100 nm oblique layer‐to‐layer misalignment on beam shaping performance.

Parameter	*δc =* 0 nm	*δc =* 25 nm	*δc =* 50 nm	*δc =* 75 nm	*δc =* 100 nm
CSR (dB)	8.89	8.96	9.13	9.58	10.40
CV (%)	26.87	27.07	27.56	27.93	28.22
DE (%)	60.23	59.69	62.01	64.07	63.71
AE (%)	10.76	10.41	10.13	9.31	6.35

It can be seen that all three misalignment have the same impact on the resulted shaped beam. As the misalignment increases, the CV gradually increases and the absolute efficiency gradually decreases. Although this represents a decrease in the uniformity and energy of the shaped beam, the changes are relatively small. Among them, oblique misalignment has the most significant impact on performance. When *δc* = 100 nm, CV increases by 1.35% and absolute efficiency decreases by 4.41%. In addition, it can be observed that CSR and diffractive efficiency have an upward trend with increasing misalignment. We speculate that this is due to layer‐to‐layer misalignment leading to an increase in unwanted reflections, resulting in a decrease in the total energy detected by the output target plane. Therefore, the calculated values of CSR and diffractive efficiency increase while the calculated values of absolute efficiency decrease, which cannot be considered as performance improvement. Overall, within the 0–100 nm misalignment range, the degradation of optical performance is within acceptable limits.

In addition, we also investigated the effect of layer‐to‐layer misalignment on the mechanical stability of the proposed structure. We simulated the stress and deformation of the free‐standing bilayer structure under gravity for three types of layer‐to‐layer misalignment reaching 100 nm. The results are shown in Figure 8, where the radius of the top layer cylindrical nanopillar is 275 nm.

**FIGURE 8:**
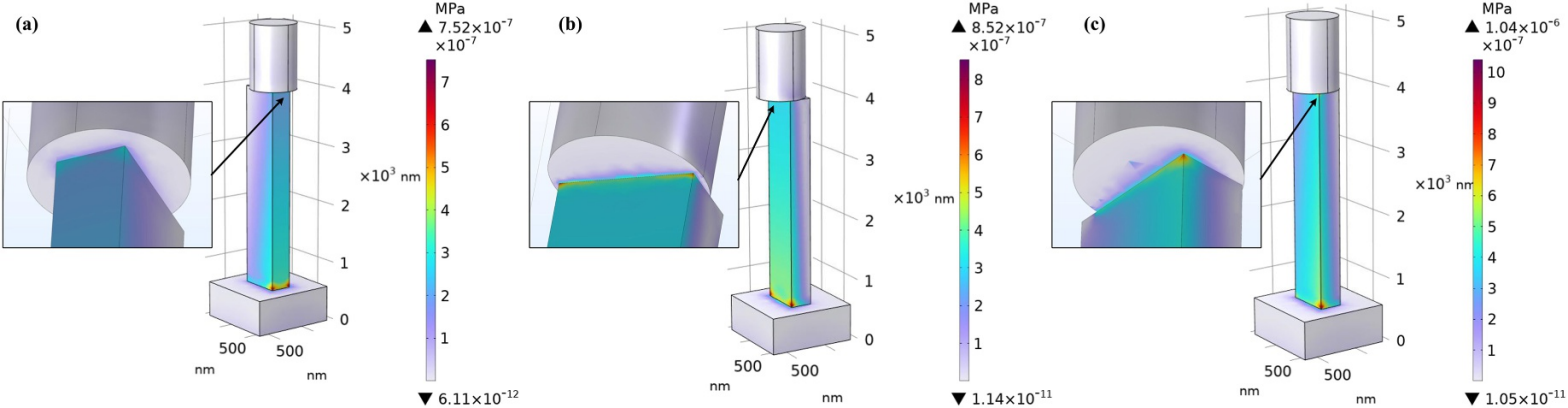
Mechanical stability analysis of free‐standing bilayer metasurfaces structures under lateral (a), longitudinal (b), and oblique (c) layer‐to‐layer misalignment.

It can be seen that layer‐to‐layer misalignment increases the overhanging part of the top layer cylindrical nanopillar, which leads to an increase in local stress on the rectangular nanopillar in the misalignment direction. The oblique misalignment has the greatest impact on mechanical stability. When the oblique misalignment reaches 100 nm, the local stress on the rectangular nanopillar increases to 1.04 × 10^−6^ MPa. However, this value is much lower than the fracture strength of TiO_2_ nanostructures, which is 0.3–4.2 GPa [22]. Therefore, the proposed structure can still maintain sufficient mechanical stability within the layer‐to‐layer misalignment range of 100 nm. In addition, we also analyzed the two key fabricating errors of sidewall taper and lateral dimensional deviation, which are described in detail in Supporting Information S1: Section S5.

### 3.4 Free‐Standing Bilayer Metasurfaces for Low Crosstalk Dual‐Channel Hologram

To further verify the dual‐channel performance of the proposed free‐standing bilayer metasurfaces, we designed it for dual‐channel hologram generation. As shown in Figure 9a, when the incident beam is LCP, the output RCP produces a lightning pattern on the target plane; when the incident beam is RCP, the output LCP produces a snowflake pattern. The traditional single‐layer spin‐decoupled metasurfaces from Supporting Information S1: Section S1 were also used as a comparison.

**FIGURE 9:**
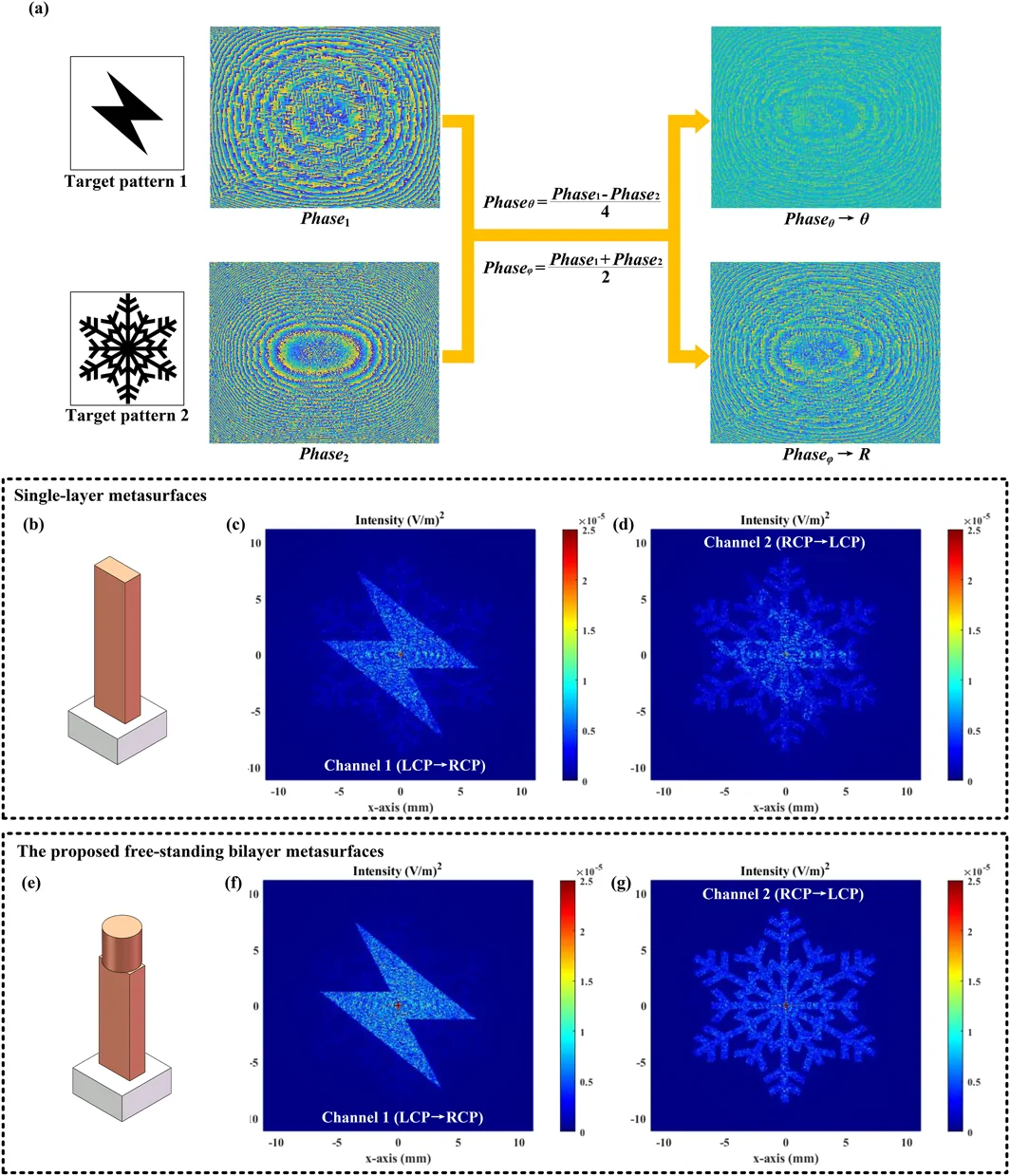
(a) Calculation process of the phases for dual‐channel hologram. (b)–(d) The dual‐channel hologram achieved by traditional single‐layer spin‐decoupled metasurfaces. (e)–(g) The dual‐channel hologram achieved by the proposed free‐standing bilayer metasurfaces.

Figure 9b‐g show the dual‐channel hologram performance of the two metasurfaces. It can be seen that the holograms generated by the traditional single‐layer spin‐decoupled metasurfaces suffer from obvious crosstalk, resulting in reduced resolution and uneven intensity in each channel. In contrast, the proposed free‐standing bilayer metasurfaces exhibits excellent performance, with clear hologram patterns in both channels.

We also calculated the evaluation values of the dual‐channel holograms achieved by two types of metasurfaces, as shown in the Table 8. Here we specifically introduce structure similarity index measure (SSIM) to compare the quality difference between the generated hologram and the target image, in order to evaluate the fidelity of the hologram. SSIM is calculated using the following equation

(8) $$\text{SSIM}\left(m,n\right)=\frac{\left(2\mu_{m}\mu_{n}+C_{1}\right)\left(2\sigma_{mn}+C_{2}\right)}{\left(\mu_{m}^{2}+\mu_{n}^{2}+C_{1}\right)\left(\sigma_{m}^{2}+\sigma_{n}^{2}+C_{2}\right)}$$

where *μ*_*m*_ and *μ*_*n*_ are the mean brightness values of images *m* and *n*, *σ*_*m*_ and *σ*_*n*_ are the standard deviation of brightness values of images *m* and *n*. *σ*_*mn*_ is the covariance of brightness values of images *m* and *n*. *C*_1_ and *C*_2_ are constants.

**TABLE 8:** Comparison of hologram quality in dual‐channel between two types of metasurfaces.

Method	Channel 1 (LCP → RCP)				Channel 2 (RCP → LCP)			
	CSR (dB)	SSIM	DE (%)	AE (%)	CSR (dB)	SSIM	DE (%)	AE (%)
The proposed metasurfaces	10.91	0.8686574	81.41	14.89	16.55	0.7091024	86.13	16.21
Single‐layer metasurfaces	6.18	0.8686570	60.13	9.34	9.32	0.7091021	67.60	10.58

It can be seen that the proposed method only slightly improves the SSIM value of holograms compared to traditional single‐layer metasurfaces. This is because SSIM mainly measures the structural similarity between reconstructed images and target images, and both designs successfully constrain most of the energy within the target shape. However, it is important to note that the proposed method achieves significantly improved CSR, diffractive efficiency, and absolute efficiency, which directly indicates that the free‐standing bilayer metasurfaces achieves better channel isolation and higher energy utilization without sacrificing images quality. In Channel 1, CSR increased from 6.18 to 10.91 dB, diffractive efficiency increased by 21.28%, and absolute efficiency increased by 5.55%. In Channel 2, CSR increased from 9.32 to 16.55 dB, diffractive efficiency increased by 18.53%, and absolute efficiency increased by 5.63%. In summary, the advantages of the proposed free‐standing bilayer metasurfaces for achieving dual‐channel hologram should be understood as significantly improving channel isolation and energy utilization while maintaining image fidelity.

## 4 Conclusion

In this paper, we proposed a free‐standing bilayer metasurfaces that achieves low crosstalk and improved efficiency in dual‐channel polarization multiplexing compared to single‐layer spin‐decoupled metasurfaces. By vertically separating geometric phase and propagation phase modulation into two independent layers, we overcome the parametric degeneracy and nonuniform polarization conversion rate that plague traditional single‐layer spin‐decoupled designs. Numerical simulations confirmed that our design significantly outperforms the single‐layer metasurfaces in dual‐channel beam shaping: the CSR from 3.81 to 8.89 dB in Channel 1, and from 3.81 to 10.72 dB in Channel 2, respectively. At the same time, both CV and efficiency are improved. This proves that the distortion caused by crosstalk is significantly weakened. The proposed metasurfaces maintains stable operation across 532 ± 30 nm. In addition, this device also maintain sufficient mechanical stability within the layer‐to‐layer misalignment range of 0–100 nm, and the degradation in optical performance is within an acceptable range. Dual‐channel hologram further corroborates the superior performance, with our device producing clear patterns with low crosstalk. Although fabrication complexity exceeds that of single‐layer devices, recent advances in multi‐step lithography and deposition make this approach increasingly feasible. This method will open up new avenues for high fidelity polarization multiplexed meta‐optics in high capacity displays, polarization imaging, and optical encryption.

## Author Contributions

**Bowen Niu:** writing – original draft, software, formal analysis, writing – review and editing, visualization, data curation. **Qun Dai:** investigation. **Yu Ao:** validation. **Xuanwei Xu:** supervision. **Yu Qiao:** conceptualization. **Yanjun Hu:** methodology, resources. **Guofang Fan:** methodology, funding acquisition, project administration.

## Funding

This study was supported by National Key R&D Program of China (Grant 2023YFF0720301).

## Supporting information


Supporting Information S1

## Data Availability

Data underlying the results presented in this paper are not publicly available at this time but may be obtained from the authors upon reasonable request.
