# Supplementary material for: Free‐Standing Bilayer Metasurface With Physically Separated Phase Modulations for Low‐Crosstalk Polarization Multiplexing
Source: Nanophotonics. 2026 Nov 11;15(22):e70368. doi: 10.1002/nap2.70368 (PMC13755746; doi:10.1002/nap2.70368)
Supplement: Supplementary file 1 [file NAP2-15-e70368-s001.pdf]

Supporting Information

Free-standing bilayer metasurface with physically separated phase modulations for low crosstalk polarization multiplexing

*Bowen Niu, Qun Dai, Yu Ao, Xuanwei Xu, Yu Qiao, Yanjun Hu, and Guofang Fan**

S1. Structure parameters of single-layer spin-decoupled metasurfaces meta-atoms.

In order to determine the structural parameters of single-layer spin-decoupled meta-atoms, a 532nm wavelength LCP beam was used as the incident beam to perform parameter scanning on rectangular nanopillars (length *L* and width *W*), with the same scanning density as the parameter scanning density of the cylindrical nanopillars radius *R* in the proposed bilayer structure. In practical simulations, it has been found that if a single-layer structure wants to achieve complete 0-2π propagation phase coverage by changing the size of the rectangular nanopillars, its polarization conversion rate often fluctuates significantly between different sizes, making it difficult to ensure both consistency and high values of polarization conversion rate at the same time. Therefore, the size of the rectangular nanopillars we ultimately selected was based on meeting the primary condition of 0-2π propagation phase coverage, and we tried to choose the optimal structure for polarization conversion rate as much as possible. It should be noted that both structures adopt the same meta-atoms library of 16-level size parameters×16-level rotation angles, and are constructed through independent and equally rigorous parameter scanning and screening processes under the same design objectives and constraints.

From the simulation set, 16 meta-atoms were selected, and their structure parameters are shown in **Table S1**. **Figure S1** shows their polarization conversion rate and propagation phase.

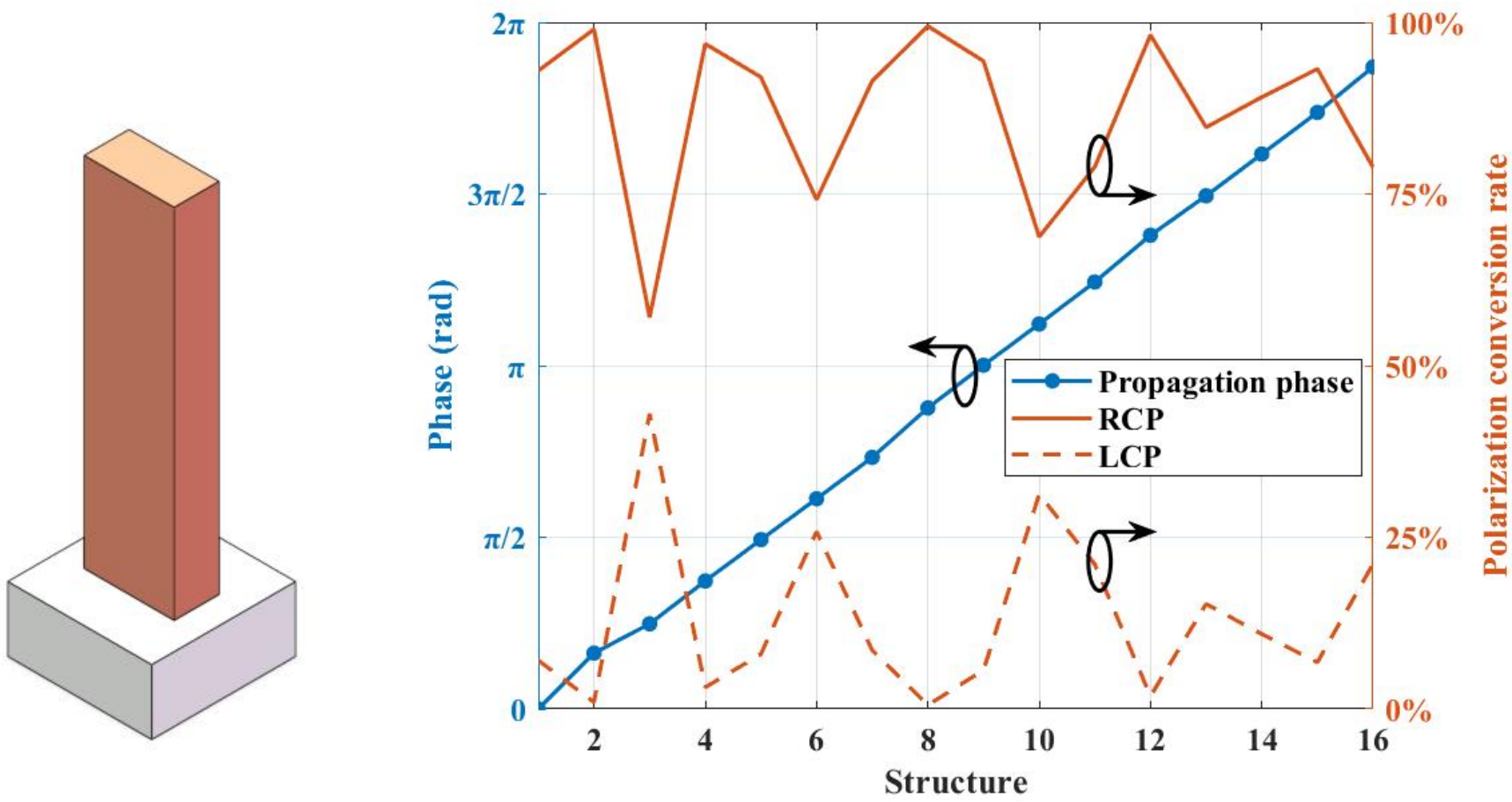

Figure S1. The polarization conversion rate and the propagation phase of the 16 selected meta-atoms.

Table S1. The structure parameters of the single-layer spin-decoupled meta-atoms.

	1	2	3	4	5	6	7	8
W(nm)	144	256	300	256	186	153	256	265
L(nm)	446	376	386	395	437	456	456	405

	9	10	11	12	13	14	15	16
W(nm)	278	125	125	278	284	113	287	116
L(nm)	405	395	414	487	433	427	494	494

S2. Phase modulation mechanism of cylindrical nanopillar.

In the proposed free-standing bilayer structure, light mainly propagates in guided wave mode inside the cylindrical nanopillar. The effective refractive index n_{eff} increases with the increase of radius R, therefore the propagation phase ($\varphi = n_{eff} \cdot k_0 \cdot H$) monotonically changes with R.

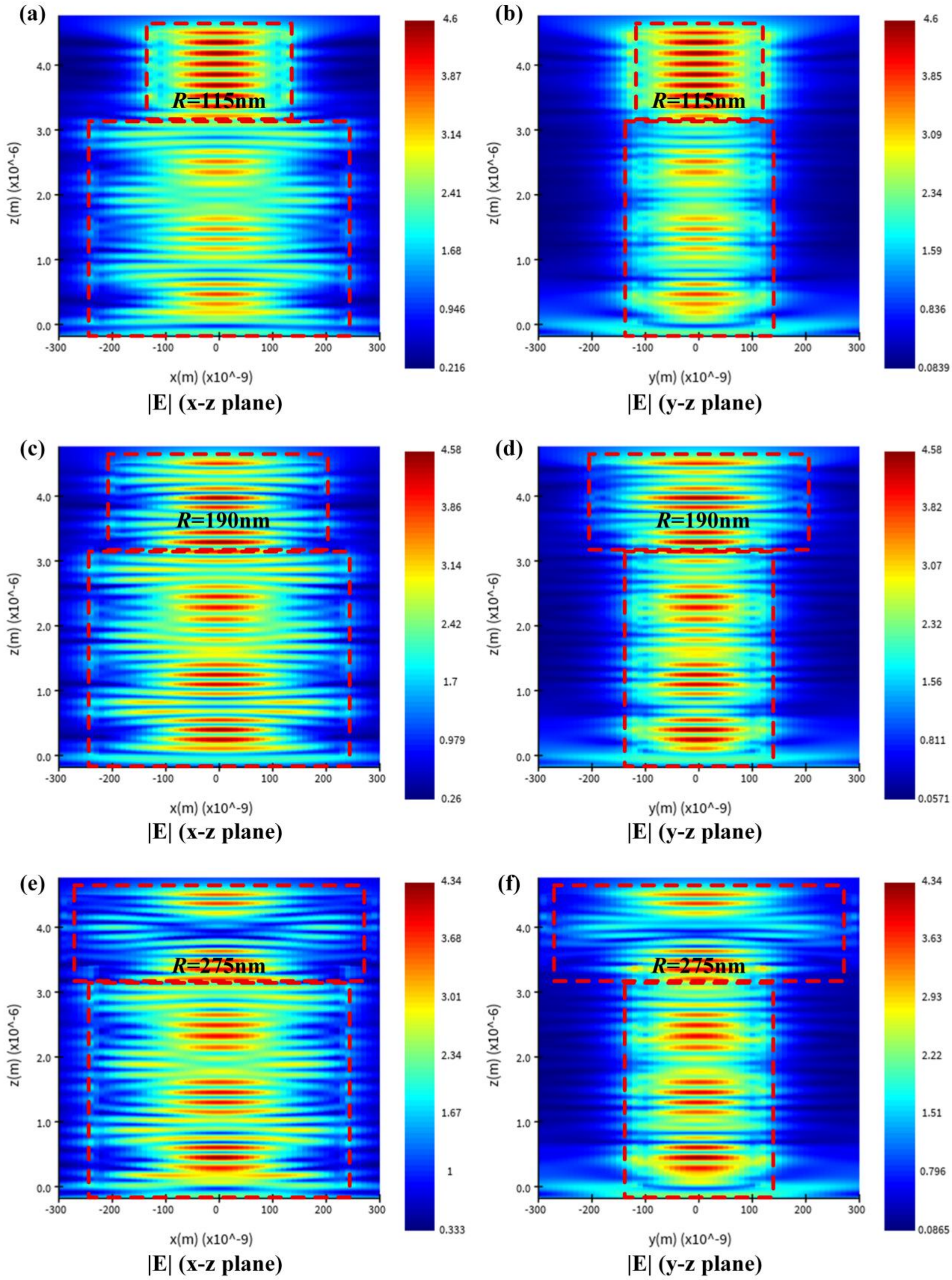

Figure S2. Electric field magnitude of the meta-atoms with different cylindrical nanopillar radii.

We calculated the electric field magnitude inside the meta-atom structure at three different cylindrical nanopillar radii of 115nm, 190nm, and 275nm, as shown in **Figure S2**. It can be seen that within the range of *R*=115-275nm, the electric field is mainly concentrated inside the TiO_2 nanopillars, exhibiting a Fabry-Pérot standing wave mode along the height direction, and the electric field rapidly decays at the nanopillars boundary. This indicates that the structure is located in the waveguide propagation region far from Mie resonance at a working wavelength of 532nm, and the phase change mainly comes from the accumulation of propagation phase caused by the effective refractive index changing with radius. In addition, cylindrical nanopillars are isotropic (circularly symmetric), and their polarization response does not significantly change with radius. Therefore, *R* controls the propagation phase without changing the polarization conversion rate, and the isotropy of the top cylindrical nanopillars ensures the same phase delay for LCP and RCP.

S3. Simulation of representative heterogeneous-supercell.

To verify that the unit-cell response of periodic boundary conditions is still valid in assembled (non-periodic) metasurface, we conducted representative heterogeneous-supercell simulation. We selected a 3×3 adjacent units block from the 1500×1500 metasurfaces structure shown in Section 3.1 for full wave simulation, which has different combinations of R and θ. And compare it with the combined results of independent unit-cell responses under periodic boundary conditions. The transmission phase responses of the two methods are shown in **Figure S3**, and the results indicate that they are relatively similar. This proves that the simulated periodic independent unit-cell response is still effective in the assembled metasurfaces.

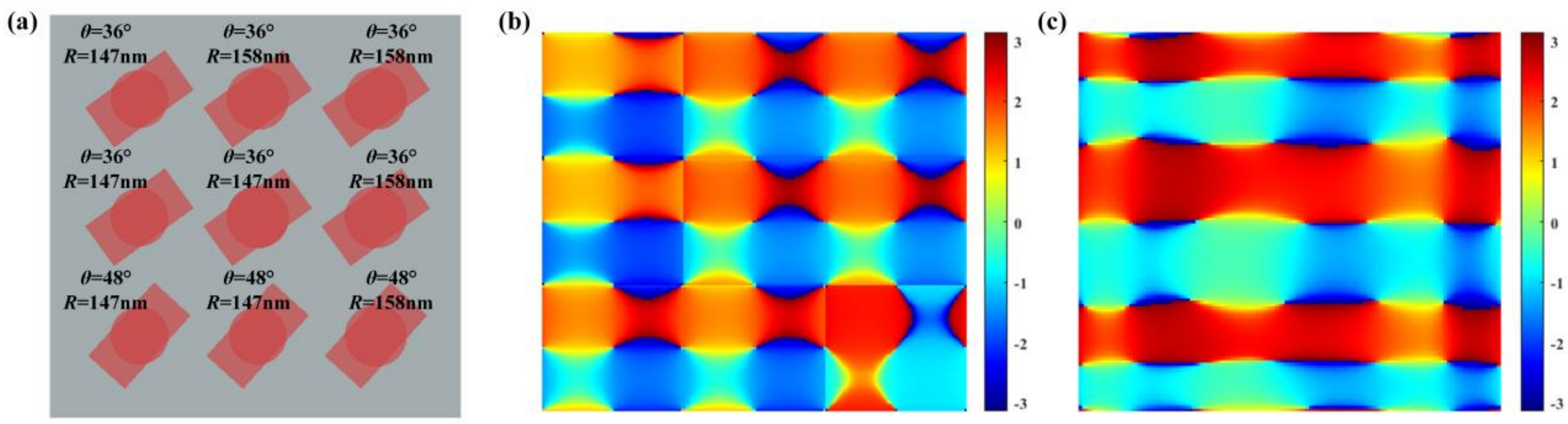

Figure S3. (a) The selected 3×3 heterogeneous-supercell from the 1500×1500 metasurface structure. (b) The transmission phase response of the combined results of independent unit-cell responses under periodic boundary conditions. (c) The transmission phase response of the heterogeneous-supercell full wave simulation.

This is because in our actual 1500×1500 device, the target phase distribution is calculated through IFTA. The IFTA itself has low-pass filtering characteristics, and the phase changes between adjacent pixels in the generated phase distribution are usually smooth. In addition, the phase distribution has undergone high-level quantization of 16-level, which together ensure that the dimensional changes of adjacent units are gradual. Therefore, simulating the local period approximation of the unit-cell is effective, and uncertain near-field coupling effects can be controlled at a lower level.

S4. The impact of 16-level phase discretization on crosstalk and *CV*.

To separately verify the impact of phase quantization, we conducted comparative simulations without considering structuring the phase distribution. We compared the *CSR* and *CV* of the shaped beams under continuous phase and 16-level discretized phase. The results are shown in the **Table S4.**

Table S4. Comparison of *CSR* and *CV* achieved by continuous phase and 16-level phase.

Method	Channel 1 (LCP→RCP)		Channel 2 (RCP→LCP)	
	CSR	*CV*	*CSR*	*CV*
Continuous phase	11.73dB	14.36%	11.73dB	14.21%
16-level phase	10.43dB	14.91%	10.68dB	14.95%

It can be seen that compared to continuous phase, discretization only leads to a degradation of CV of less than 1%, and the change in crosstalk is also relatively small. This is mainly due to the high quantization level of 16, and this high-level phase discretization is not the main source of crosstalk, with little impact on performance.

S5. The influences of sidewall taper and dimensional deviation on the working performance.

To further evaluate the practical fabricating limitations of the proposed free-standing bilayer metasurfaces, we simulated the influences of sidewall taper and dimensional deviation on the working performance. Here we used the dual-channel beam shaping metasurfaces described in Section 3.1, taking the incident LCP beam as an example.

The sidewall taper is a typical non ideal situation in practical fabricating of high-aspect-ratio structures. Due to the significantly higher height of the bottom rectangular nanopillars compared to the top cylindrical nanopillars in the proposed free-standing bilayer structure, the sidewall taper of the bottom rectangular nanopillars will be more pronounced. Therefore, we mainly consider the impact of the sidewall angle θ_{ST} of the bottom rectangular nanopillars on the metasurfaces working performance. Due to the possible existence of two types of taper shapes: regular taper (θ_{ST}<90°) and inverted taper (θ_{ST}>90°), as shown in **Figure S5(a)**. We discussed the θ_{ST} at 90±2°, the results are shown in **Table S5**. Assuming that the taper exists on both the long and wide sidewalls of the rectangular nanopillars.

The results indicate that when θ_{ST}<90°, the metasurfaces performance significantly deteriorates, but when θ_{ST}>90°, the metasurfaces performance slightly improves. For a regular taper with θ_{ST}<90°, the cross-section of the bottom rectangular nanopillars change relative to the design value, which may cause the nanopillars to deviate from the optimal half-wave condition and reduce the polarization conversion rate. This may further distort the geometric phase response and increase crosstalk. On the contrary, for the inverted taper with θ_{ST}>90°, although it may also deviate from the half-wave condition, the profile provides a gradient equivalent refractive index transition, similar to the taper coupling structure at the interface of the optical waveguide. Therefore, it may bring a slight performance improvement. It should be emphasized that the performance improvement under the inverted taper should not be interpreted as an advantage of the sidewall taper. We still hope to maintain the sidewall angle at the design value of 90°.

It should be noted that the free-standing bilayer structure proposed in this paper is based on the validated fabrication framework in Dorrah et al.' s work in 2025 (Ref. 20), which is a filling process similar to the Damascene. Firstly, it is necessary to use electron beam lithography to create "holes" in the photoresist layer. Then, through deposition technology, TiO_2 is filled into these "holes", and finally the photoresist is removed, leaving TiO_2 nanopillars. The key is that the shape of the "holes" in the photoresist will be directly reverse engraved into the shape of TiO_2 nanopillars. Due to the process characteristics of exposure and development, the bottom

of the "holes" in the photoresist layer may be narrower than the top (i.e., the "holes" form an inverted taper), and the final filled TiO_2 nanopillars will naturally have a wider top than bottom. Therefore, θ_{ST}>90° is more likely to occur in actual processing situations.

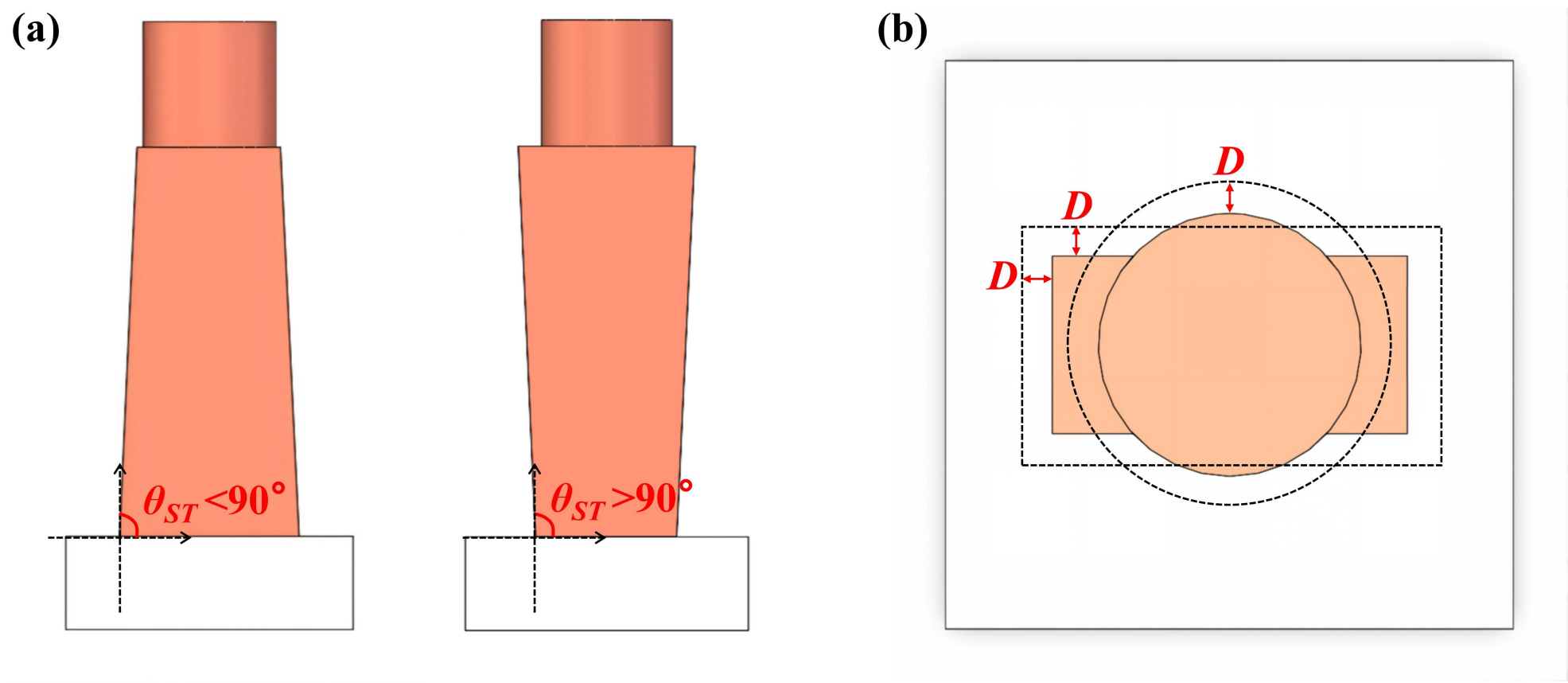

Figure S5. Schematic diagram of sidewall taper (a), and lateral dimensional deviation (b).

Table S5. The influences of 90±2° sidewall angle on working performance.

Parameter	θ_{ST}=88°	θ_{ST}=89°	θ_{ST}=90°	θ_{ST}=91°	θ_{ST}=92°
CSR	7.66dB	8.87dB	8.89dB	9.18dB	9.74dB
CV	30.57%	27.46%	26.87%	25.08%	24.59%
DE	51.26%	55.72%	60.23%	62.17%	66.32%
AE	2.78%	5.36%	10.76%	11.85%	14.43%

Table S6. The influences of ±20nm lateral dimensional deviation on working performance.

Parameter	*D*=-20nm	*D*=-10nm	*D*=0nm	*D*=+10nm	*D*=+20nm
CSR	5.16dB	7.69dB	8.89dB	7.25dB	4.94dB
CV	34.71%	29.29%	26.87%	29.12%	33.45%
DE	53.32%	57.55%	60.23%	57.90%	52.97%
AE	4.53%	9.62%	10.76%	9.33%	4.08%

In addition, in terms of dimensional tolerances, we evaluated the influence of the lateral dimensional deviation *D* at ±20nm, as shown in **Figure S5(b)**. Assuming that the lateral dimension deviation exists simultaneously in both the bottom rectangular nanopillars and the top cylindrical nanopillars, the results of the metasurfaces working performance are shown in **Table S6**.

The simulation results indicate that as the lateral dimension deviation of the structure increases, the working performance of the metasurfaces will significantly deteriorate. Among them, the radius deviation of the top cylindrical nanopillars directly shifts the propagation phase, which is the main source of phase error. The length and width deviation of the bottom rectangular nanopillars mainly affect their half-wave conditions, thereby changing the

polarization conversion rate. We suggest controlling the lateral dimension deviation within ±10nm during processing to ensure that the performance remains within an acceptable range.
